# Comparative Analysis of Viral Load and Cytokines during SARS-CoV-2 Infection between Pregnant and Non-Pregnant Women

**DOI:** 10.3390/ijms25147731

**Published:** 2024-07-15

**Authors:** Dakai Liu, Hui Li, Xiaofeng Li, George D. Rodriguez, Harlan Pietz, Roberto Hurtado Fiel, Eric Konadu, Vishnu Singh, Florence Loo, William Harry Rodgers

**Affiliations:** 1 Department of Pathology and Clinical Laboratories, NewYork-Presbyterian Queens, 56-45 Main Street Flushing, New York, NY 11355, USA; 2 National Clinical Research Center for Respiratory Disease, State Key Laboratory of Respiratory Disease, Guangzhou Medical University, Guangzhou 510182, China; 3 NewYork-Presbyterian Queens, 56-45 Main Street Flushing, New York, NY 11355, USA; 4 DMC Detroit Receiving Hospital, 4201 St Antoine, Detroit, MI 48201, USA; 5 Department of Pathology and Laboratory Medicine, Weil Cornell Medical College, 1300 York Avenue, New York, NY 10065, USA

**Keywords:** SARS-CoV-2, infection, pregnant, non-pregnant, viral load, cytokine

## Abstract

To better understand the vulnerabilities of pregnant women during the COVID-19 pandemic, we conducted a comprehensive, retrospective cohort study to assess differences in immune responses to SARS-CoV-2 infection between pregnant and non-pregnant women. Nasopharyngeal swabs and serum specimens from 90 pregnant and 278 age-matched non-pregnant women were collected from 15 March 2020 to 23 July 2021 at NewYork-Presbyterian Queens Hospital in New York City. Multiplex reverse transcription polymerase chain reaction, neutralizing antibody, and cytokine array assays were used to assess the incidence, viral load, antibody titers and profiles, and examine cytokine expression patterns. Our results show a lower incidence of SARS-CoV-2 infection in pregnant women compared with non-pregnant women. Pregnant women infected with SARS-CoV-2 exhibited a substantially lower viral load. In addition, the levels of both anti-spike protein receptor-binding domain IgG neutralizing antibodies and anti-N Protein IgG were elevated in pregnant women. Finally, cytokine profiling revealed differential expression of leptin across cohorts. These findings suggest that pregnancy is associated with distinct immune and virological responses to SARS-CoV-2 infection, characterized by lower infection rates, substantially lower viral loads, and enhanced antibody production. Differential cytokine expression indicates unique immune modulation in pregnant women.

## 1. Introduction

The Coronavirus Disease 2019 (COVID-19) pandemic has had a disproportionate impact on certain populations, including pregnant women [1]. As of April 2024, the global case count had exceeded 775 million, with over 7 million fatalities [2,3]. The immune responses to Severe Acute Respiratory Syndrome Coronavirus 2 (SARS-CoV-2), the causative agent of COVID-19, are complex and poorly understood, especially among pregnant women [3,4].

The prevailing view that pregnancy induces a form of immune compromise has prompted concerns about heightened susceptibility to viral pathogens [5,6]. This is partly because pregnancy involves immune adaptations that prevent maternal rejection of the fetus, treated immunologically as a semi-allogenic graft [7]. However, recent studies have suggested that the immune responses during pregnancy are far more nuanced [8,9]. These observations raise questions about the actual risk pregnant women face regarding SARS-CoV-2 infection and the potential impact on maternal and fetal outcomes.

Diagnostic tools are pivotal for understanding not just the presence of the virus but also the underlying immune responses and their divergence among pregnant women. By understanding diagnostic metrics in pregnant women compared to their non-pregnant counterparts, we aim to identify unique protections or vulnerabilities conferred by pregnancy against SARS-CoV-2 infection.

## 2. Results

### 2.1. The Incidence of SARS-CoV-2 Infection in Pregnant Women Is Lower than in Age-Matched, Non-Pregnant Women

From an epidemiological perspective, the prevalence of SARS-CoV-2 infection is gauged using the incidence proportion [10]. We compared the incidence proportion of SARS-CoV-2 infection between pregnant women and non-pregnant women within the same age bracket (18–49 years old). Out of the 3078 pregnant women tested, 125 tested positive for SARS-CoV-2 infection via RT-PCR (4.1%). In contrast, of the 8716 non-pregnant women, 1003 (11.5%) tested positive. We found this difference to be statistically significant (*p* value < 0.0001 by Pearson chi-square).

### 2.2. The RT-PCR Ct Value of SARS-CoV-2-Positive Pregnant Women Is Higher than in Age-Matched, Non-Pregnant Women

**Figure 1:**
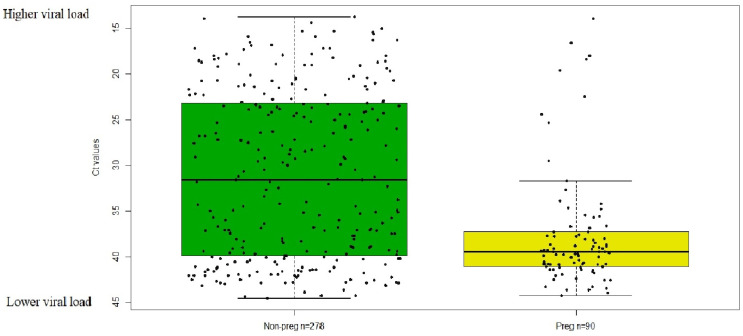
The Ct values of pregnant and non-pregnant women. The median Ct value in pregnant women is significantly higher than in non-pregnant women (*p* value < 0.001).

The median Ct value of nasopharyngeal swab samples from 90 pregnant women (39.5) was significantly higher (*p* < 0.001), indicating a lower viral load, than that from 278 non-pregnant women (31.6) (Figure 1). Additionally, the interquartile range was greater among non-pregnant women (16.7, upper quartiles = 39.9, lower quartiles = 23.2) than among pregnant women (3.8, upper quartiles = 41.1, lower quartiles = 37.3), even though the highest and lowest Ct values were comparable (pregnant highest 44.3, non-pregnant highest 44.6; and pregnant lowest = 13.7 and non-pregnant lowest = 13.9).

The data revealed that the mean viral load, as indicated by the N2 gene’s Ct value/target Ct value [11,12,13], was appreciably lower in the 90 pregnant women specimens (37.7) than the 278 non-pregnant women specimens (31.1). The Mann–Whitney U test was performed to compare the mean of non-normally distributed Ct values in pregnant women and age-matched non-pregnant women (z = −5.7125, *p* = 3.8 × 10^−8^). The difference in Ct values between pregnant women and non-pregnant women (6.6) suggests that pregnant women infected with SARS-CoV-2 might bear a viral load that is 97 times less than non-pregnant women.

### 2.3. Measurement of SARS-CoV-2 Neutralizing Antibody (nAb)

**Figure 2:**
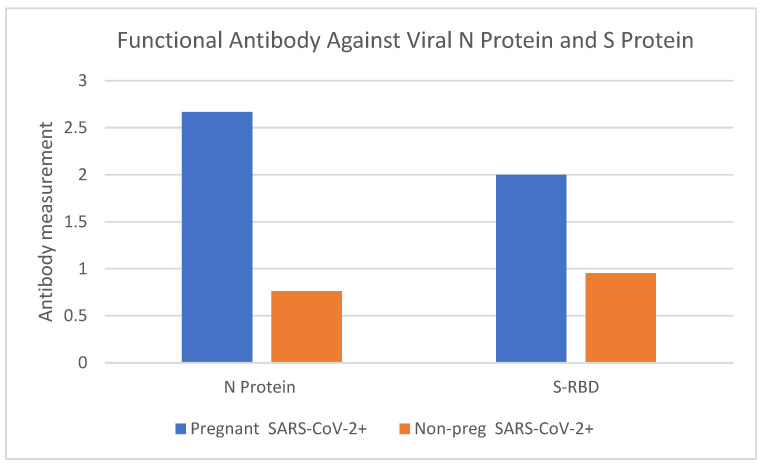
Mean S-RBD neutralizing IgG levels and the N protein IgG in pregnant women infected with SARS-CoV-2+ and non-pregnant women infected with SARS-CoV-2+.

We retrospectively measured and compared SARS-CoV-2 S-RBD IgG neutralizing antibody and N Protein IgG antibody between a randomly available subset of six pregnant and twenty-one non-pregnant SARS-CoV-2-positive patient specimens. Both the mean SARS-CoV-2 S-RBD IgG neutralizing antibody levels and mean N Protein IgG antibody levels in pregnant women were higher than in their non-pregnant counterparts (Figure 2).

### 2.4. Leptin Cytokine Profiling Reveals Differential Expression across Cohorts

**Figure 3:**
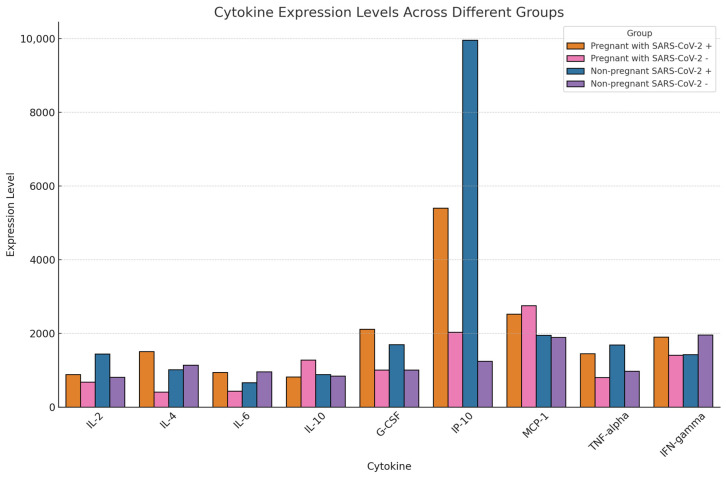
The expression levels of nine cytokines in four groups. Values are expressed as cytokine array relative pixel density units.

Cytokines, chemokines, and growth factors are critical elements in both beneficial and pathological immune responses against viral infections. In particular, diseases can trigger a cytokine storm, in which an excessive number of cytokines are released, inducing high levels of inflammation. The levels of ten cytokines associated with COVID-19 cytokine storm are presented in Table 1 as well as Figure 3.

**Figure 4:**
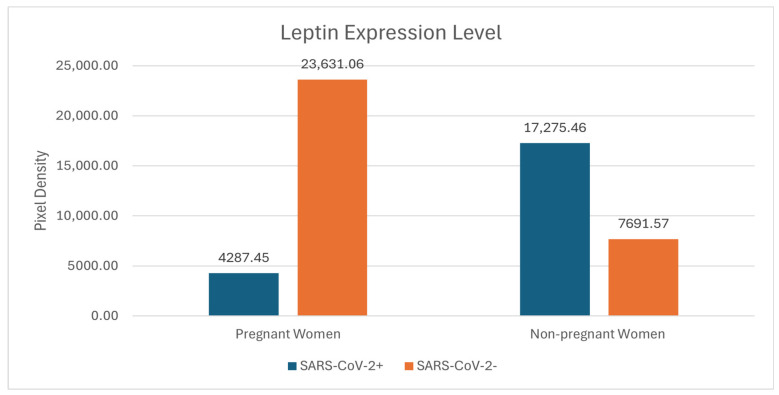
The leptin expression level reading across the four groups.

Among the cytokines, leptin showcased distinct expression profiles across the four cohorts (Figure 4). Leptin showed a higher value in pregnant women, regardless of their SARS-CoV-2 status than in non-pregnant women.

## 3. Discussion

The study of COVID-19 has unveiled a myriad of physiological responses across different demographics. Among the most intriguing are the differential responses between pregnant and non-pregnant women. Our findings provide insights into this disparity and shed light on the potential protective mechanisms at play during pregnancy.

The significantly lower SARS-CoV-2 positivity rate in pregnant women, compared with age-matched non-pregnant women, is noteworthy. This observation, coupled with the finding that pregnant women also harbor a markedly lower viral load as denoted by higher Ct values, might suggest an innate resistance to SARS-CoV-2 infection. Innate immunity includes (1) anatomical barriers including skin and mucous membranes, (2) intact normal flora, (3) soluble proteins, which include complement, acute phase proteins, and interferons, and (4) the ability to induce inflammatory and phagocytic response. The Ct value does not provide an absolute quantification but offers insight into viral load. Viral load may serve as a predictor of both contagiousness and disease severity [14] since it quantifies the concentration of the virus present in an infected individual [11,15,16]. Typically, Ct values oscillate between 15 and 45 cycles. A higher viral presence necessitates fewer cycles to reach the detection threshold, translating to a lower Ct value. Thus, a lower Ct value is inversely associated with the viral load in a sample.

Among the pregnant women, nine cases were found with a Ct value lower than the Boxplot-defined minimum value (pregnant 31.7, Figure 1). Five out of the nine cases were in clusters located in adjacent zip codes between April 3rd and April 10th, 2020. This would warrant further investigation of molecular epidemiology if any variant is linked to these clusters. Another four out of the nine cases had underlying medical conditions including diabetes, heart disorders, anemia, and neurologic diseases.

The severity and mortality of COVID-19 are often attributed to a cytokine storm [17]. Of the 105 cytokines profiled in this study, nine were previously reported to be involved in the cytokine storm associated with COVID-19 including the following: IL-2, IL-4, IL-6, IL-10, G-CSF, IP-10, MCP-1, TNF-α, and IFN-γ [18,19,20]. The expression levels of these cytokines across the four cohorts are detailed in Table 1 and Figure 3.

Leptin serves various roles in immunity, energy homeostasis, and reproductive functions. Recent investigations into the biological determinants of severe COVID-19 manifestations have spotlighted leptin, a plasma protein, as a significant factor. Elevated leptin levels have been identified in both mild and severe cases of COVID-19, and its prognostic capability has surpassed even that of traditional inflammation markers like IL-6 and CXCL-101 [21]. Leptin also orchestrates a variety of immune responses, including regulating cytokine, chemokine, and interferon production, altering the Th1/Th2 balance, and adjusting macrophage phagocytic activities [22]. Given its multifaceted roles in the immune system, leptin’s involvement may significantly shape the pathophysiology of COVID-19 by influencing monocyte signaling and inflammatory reactions [23,24].

Our results show elevated leptin levels in pregnant women not infected with SARS-CoV-2 compared with non-pregnant women. This heightened leptin level suggests altered autoimmunity shielding pregnant women from SARS-CoV-2. Cytokine storm, an overexuberant immune response, has been identified as a feature of severe COVID-19 infection. The observed leptin modulation suggests a strategic adaptation during pregnancy, possibly offering protection against the virus itself and/or the severe immune reactions it can induce [17,18,19,20].

Our findings highlight elevated neutralizing antibody levels in pregnant women. Neutralizing antibodies play a pivotal role in thwarting viral infections by preventing the virus from entering host cells. Thus, a heightened level in pregnant women could be a primary factor in their diminished infection rates and reduced viral loads.

Our study is not without limitations. First, our clinical laboratory used multiple mRT-PCR SARS-CoV-2 assay devices over time, of which only Cepheid^®^ Xpert Xpress PCR provided Ct values, limiting our Ct value analysis. Second, the neutralizing antibody and cytokine testing capabilities were not available in real time, requiring retrospective analysis using available specimens, limiting our sample size and thus hampering the generalizability of our results. Third, though we excluded SARS-CoV-2 re-infection, we cannot be certain the patients included were not previously infected with asymptomatic SARS-CoV-2 infection prior to initial presentation. Fourth, our data do not include patient demographics or clinical details of each patient, including the number of days from symptom onset at the time of testing. Lastly, the analysis is purely laboratory-driven and does not include a comprehensive analysis of the patients’ clinical features, so the presence of confounding factors such as comorbidities and body mass index may limit the interpretation of the data.

Although we did not conduct preventive sample size calculations, our data suggest that pregnancy may provide protective effects against SARS-CoV-2 consisting of heightened levels of neutralizing antibodies and leptin. Understanding these dynamics provides avenues for diagnostic and therapeutic interventions. Further studies delving into these mechanisms could pave the way for innovative treatments and preventative measures against COVID-19 and perhaps other viral pathogens.

## 4. Materials and Methods

Multiplex reverse transcription polymerase chain reaction (mRT-PCR), the Hecin 2019-nCoV neutralizing antibody test, and the Proteome Profiler™ Human XL Cytokine Array were used to investigate viral infection rate, viral load, antibody levels, and cytokine profiles. Both neutralizing antibody (nAb) and cytokine profile testing were retrospectively performed using available serum from another study.

### 4.1. Study Inclusion and Exclusion Criteria

**Figure 5:**
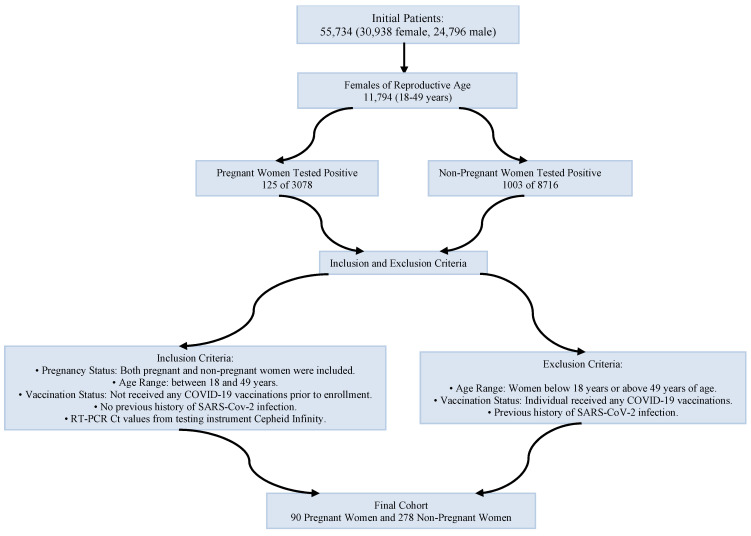
Workflow of subject selection process with inclusion and exclusion criteria.

During the period of 15 March 2020 to 23 July 2021, our clinical laboratory processed 121,706 nasopharyngeal swab specimens for SARS-CoV-2 testing. These specimens were collected from a patient pool numbering 55,734 unique individuals (30,938 female and 24,796 male). During the study period, every patient who presented to our hospital was tested for SARS-CoV-2 RNA regardless of symptoms. Among females, 11,794 were considered to be of reproductive age (18–49 years). The inclusion criteria included the following: (a) female; (b) 18 to 49; and (c) presence of an RT-PCR Ct value from the testing instrument Cepheid Infinity. Patients were excluded if they received SARS-CoV-2 vaccine or reported SARS-CoV-2 reinfection history. The pregnant individuals were admitted for labor/delivery. In total, 90 pregnant and 278 non-pregnant women met the inclusion criteria (Figure 5).

### 4.2. Detection of SARS-CoV-2 RNA

SARS-CoV-2 RNA nasopharyngeal swab specimens were assayed for SARS-CoV-2 RNA by mRT-PCR, using the Cepheid^®^ Xpert Xpress SARS-CoV-2 PCR on Cepheid^®^ Infinity. This assay consists of two amplicons with specific sets of primers/probes. Amplicon 1 targets the region in the viral nucleocapsid gene unique to SARS-CoV-2. Amplicon 2 targets a conserved region of the viral protein envelope gene homologous to all coronaviruses of the Sarbecovirus sub-genus. In addition, a sample processing control and a probe check control are also included for PCR performance. The assay analytical sensitivity was determined by serial dilutions of ZeptoMetrix virus stock—NATSARS(CoV2)-ERC with a known concentration. The limit of detection was 30 virions per assay.

Viral loads were estimated using cycle threshold (Ct) values, which are a good indicator of the amount of the virus an infected person harbors [11,12]. A Ct value denotes the number of cycles necessary for the viral RNA in a sample to surpass a predefined threshold, resulting in a positive detection [13]. A Ct value of RT-PCR testing was available only for the specimens tested on Infinity since RT-PCR performed on other instruments in our lab (such as BioFire Torch) does not provide a Ct value. Ct value variability was visualized using boxplots generated in R.

### 4.3. Detection of Anti SARS-CoV-2 Neutralizing Antibodies (nAbs)

In order to detect and measure the presence of Anti SARS-CoV-2 neutralizing antibodies (nAbs) in serum samples, Hecin 2019-nCoV Neutralizing Antibody test kits (Hecin Scientific, Inc., Guangdong, China) were used to analyze serum samples collected from 6 pregnant women and 21 non-pregnant women. Serum samples were assayed per the manufacturer’s instructions. Hecin 2019-nCoV Neutralizing Antibody test kits, which are colloidal gold immunochromatographic assay (GICA) kits, specifically detect the SARS-CoV-2 viral spike glycoprotein protein receptor-binding-domain (S-RBD) IgG and nucleocapsid (N) protein IgG antibodies.

Antibody levels were categorized into five grades, ranging from 0 to 4, as follows:

0—Presence of the Control-line with the absence of S- and N-lines.

1—A discernible but faint S- or N-line.

2—S- and/or N-line intensity is less than 50% of the Control-line intensity.

3—S- and/or N-line intensity is more than 50% but less than 100% of the Control-line intensity.

4—S- and/or N-line intensity is equal to or exceeds 100% of the Control-line intensity.

### 4.4. Cytokine Profiling

Differences in cytokine profiles between pregnant and non-pregnant women were assayed using Proteome Profiler™ Human XL Cytokine Array (R&D Systems, Inc., Minneapolis, MN, USA). Sixteen serum samples were collected from both pregnant women and non-pregnant women with and without SARS-CoV-2 infection (four samples each group). The serum samples were incubated with a Proteome Profiler™ Human XL Cytokine Array per the manufacturer’s instructions. This array was designed to simultaneously detect the relative levels of 105 cytokines, chemokines, growth factors, and other soluble proteins in a single sample. After the completion of the incubation period, the array was washed to remove unbound proteins. Detection antibodies were added to the array, followed by a chemiluminescent detection reagent. The chemiluminescent intensity of pixels was then captured using Amersham Imager 600 (GE Healthcare Life Sciences, Uppsala, Sweden). The intensity of these signals (duplicated spots for each cytokine) corresponds to the amount of cytokine bound. The acquired data were analyzed using image analysis software. The pixel density was calculated by quantifying the intensity of the signals for each cytokine and normalizing the intensity by subtracting the background signal. Comparative analysis was conducted between the pregnant and non-pregnant groups to determine differences in their cytokine profiles.

### 4.5. Statistics Analysis

Pearson’s chi-squared test was employed to analyze the incidence proportion of SARS-CoV-2 infection between pregnant women and non-pregnant women. We used a Box plot in the R language to visualize Ct value variability. The Mann–Whitney U test was performed to compare the mean of non-normally distributed Ct values in pregnant women and age-matched non-pregnant women.

## 5. Conclusions

Our study demonstrates that pregnant women infected with SARS-CoV-2 present with a substantially lower viral load, possibly from distinct immunological and virological adaptions. Our findings highlight the importance and need for further study of antibody production, immune modulation, and cytokine expression during SARS-CoV-2 infection in vulnerable populations.

## Figures and Tables

**Table 1:** Profiles of the ten cytokines implicated in the cytokine storm associated with COVID-19. Values are expressed as cytokine array relative pixel density units.

Cytokines	Pregnant SARS-CoV-2(+)	Pregnant SARS-CoV-2(−)	Non-pregnant SARS-CoV-2(+)	Non-pregnant SARS-CoV-2(−)
IL-2	886.29	685.47	1447.61	811.74
IL-4	1509.78	407.47	1015.37	1142.56
IL-6	948.03	435.63	664.66	959.9
IL-10	822.54	1281.17	885.1	845.18
G-CSF	2116.03	1006.36	1697.41	1011.39
IP-10	5401.42	2036.32	9956.05	1250.13
MCP-1	2522.03	2759.13	1955.63	1892.87
TNF-alpha	1450.14	806.3	1693.01	974.52
IFN-gamma	1902.71	1408.04	1428.87	1956.29
Leptin	4287.45	23,631.06	17,275.46	7691.57

## Data Availability

The data presented in this study are available upon request from the corresponding authors.
